# When the heart and tumours intertwine: pulmonary hypertension associated with a neuroendocrine tumour—a case report

**DOI:** 10.1093/ehjcr/ytae678

**Published:** 2024-12-31

**Authors:** Maicol Cortez, Bryam López, Julio Mamani, Flor de María Ibáñez, Gustavo Miranda

**Affiliations:** Department of Cardiology, National Hospital Edgardo Rebagliati Martins, Avenida Edgardo Rebagliati 490, Jesús María, 15072 Lima, Perú; Department of Cardiology, National Hospital Edgardo Rebagliati Martins, Avenida Edgardo Rebagliati 490, Jesús María, 15072 Lima, Perú; Department of Nuclear Medicine, Hospital Nacional Edgardo Rebagliati Martins, EsSalud, 490, Jesús María, 15072 Lima, Perú; Department of Interventional Cardiology, Hospital Nacional Edgardo Rebagliati Martins, EsSalud, 490, Jesús María, 15072 Lima, Perú; Department of Interventional Cardiology, Hospital Nacional Edgardo Rebagliati Martins, EsSalud, 490, Jesús María, 15072 Lima, Perú

**Keywords:** Heart failure, Neuroendocrine tumour, Pulmonary hypertension, Pulmonary vascular disease, Case report

## Abstract

**Background:**

Pulmonary hypertension caused by extrinsic pulmonary vascular compression secondary to mediastinal neuroendocrine tumours is a very rare condition, posing a diagnostic challenge. There is no clear consensus regarding the best treatment strategy due to the lack of clinical data, leading to poor prognoses for these patients.

**Case summary:**

We present the case of a 38-year-old man hospitalized with signs of pulmonary hypertension and acute heart failure. He had experienced progressive dyspnoea over the 12 months prior to admission. Studies performed at our institution revealed dilation of the right heart chambers with right ventricular systolic dysfunction and pulmonary hypertension. Cardiac tomography showed extrinsic vascular compression, leading to emergency endovascular treatment for superior vena cava syndrome, followed by stent implantation in the pulmonary arteries and innominate vein. Further studies identified a mediastinal neuroendocrine tumour, for which chemotherapy was initiated, without clinical response. During outpatient follow-up, cardiac function worsened, and the patient died 48 months after symptoms onset due to a lung infection.

**Discussion:**

Pulmonary hypertension secondary to extrinsic vascular compression by mediastinal tumour is a rare condition that presents a diagnostic challenge. This case highlights the importance of considering oncological aetiology in patients with progressive dyspnoea and pulmonary vascular extrinsic compression. Despite early treatment, the prognosis for these patients remains poor.

**Learning points**

- Evaluate the diagnostic approach for right heart failure, pulmonary hypertension, and mediastinal neuroendocrine tumour to provide comprehensive and personalized treatment.
- Recognize and value the importance of multidisciplinary collaboration and shared decision-making in the management of complex cases, such as heart failure, pulmonary hypertension, and malignancies.

## Introduction

Pulmonary hypertension (PH) is a progressive haemodynamic disorder that leads high mortality if not adequately treated.^1^ Pulmonary hypertension related to malignant disease can occur through complex pathophysiological mechanisms, either through direct effects of tumour microemboli, extrinsic pulmonary vascular compression, or secondary to cancer-related therapy, including chemotherapy, radiation, and haematopoietic stem cell transplantation.^2^ Pulmonary hypertension caused by extrinsic pulmonary vascular compression secondary to mediastinal tumours is a very rare cause. This type of PH fits into Group 5 of the World Health Organization’s PH classification.^3,4^

Mediastinal atypical carcinoid is a rare and aggressive type of neuroendocrine tumour (NET).^5^ Neuroendocrine tumours of the thymus are much rarer than primary carcinoids of the lung, with a reported incidence of 0.02–0.04 per 100 000 inhabitants per year.^6^ Thymic carcinoids occur primarily in men in a ratio of 3:1 to women.^7^ Exhaustive surgical removal of the thymus is the treatment of choice for thymic carcinoids.^5^ However, due to the rarity of thymic neuroendocrine neoplasms, there is a lack of clinical trials and large series, so there are few consensus statements or clear guidelines for optimal treatment.^8^

## Summary figure

**Figure:**
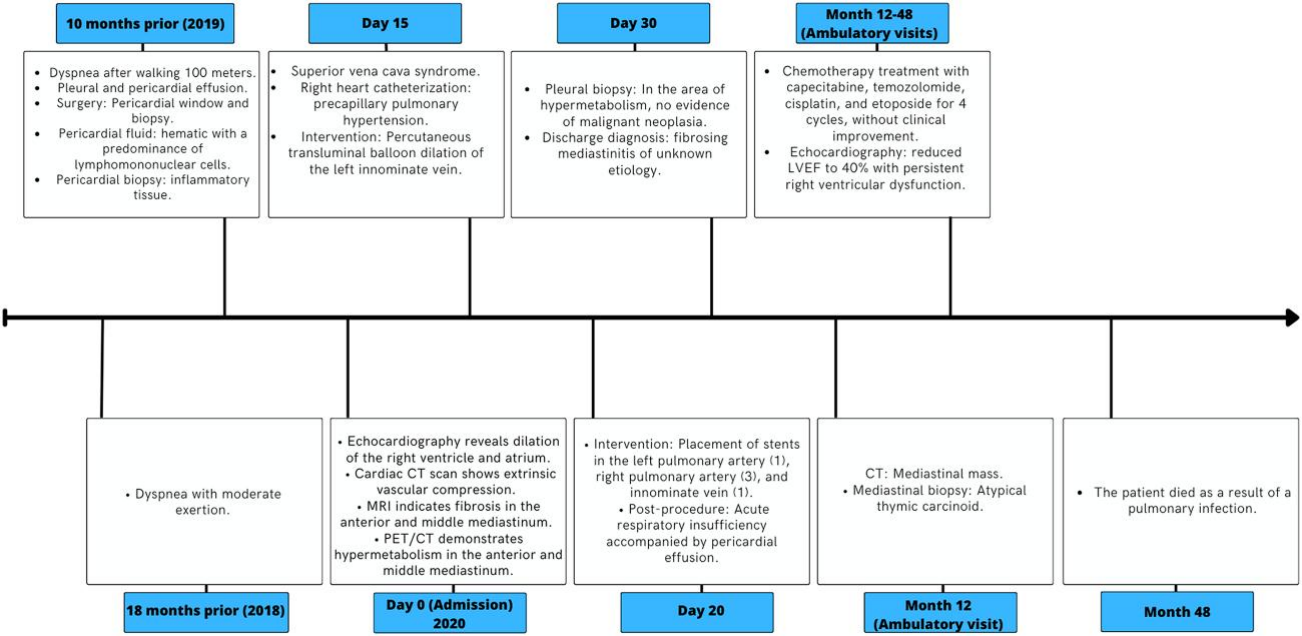


## Case presentation

A 38-year-old man with no cardiovascular risk factors was admitted to our institution for evaluation of PH and progressive dyspnoea. On admission, the patient was normotensive, heart rate 76 beats per minute, normal oxygen saturation (room air). Physical examination demonstrated the presence of oedema in the lower limbs, jugular engorgement, and hepatomegaly. Electrocardiogram (ECG) showed sinus rhythm, borderline right axis deviation (+90°), and S1Q3T3 pattern (see Supplementary material online, *Figure S1*).

The patient’s clinical history began 12 months before admission, when he started experiencing dyspnoea on moderate exertion. Over the months, the dyspnoea worsened, limiting his ability to walk distances <100 m. At the hospital from which he was referred, a severe pleural and pericardial effusion was diagnosed. Given this situation, a pericardial window and a pericardial biopsy were performed. Analysis of the pericardial fluid revealed haematic characteristics, with a predominance of lymphomononuclear cells. Inflammatory tissue was observed in the pericardial biopsy, with no evidence of oncological pathology. The initial differential diagnosis considered heart failure with PH, biventricular dysfunction, and chronic pulmonary thromboembolism.

**Figure 1:**
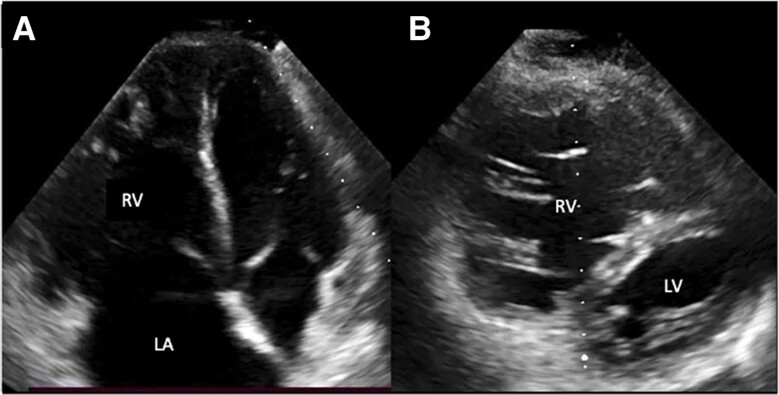
Transthoracic echocardiography. (*A*) Apical four-chamber view shows marked dilation of the right atrium (LA) and right ventricle (RV). (*B*) Short-axis view shows dilation of the right ventricle and flattening of the interventricular septum in systole.

**Figure 2:**
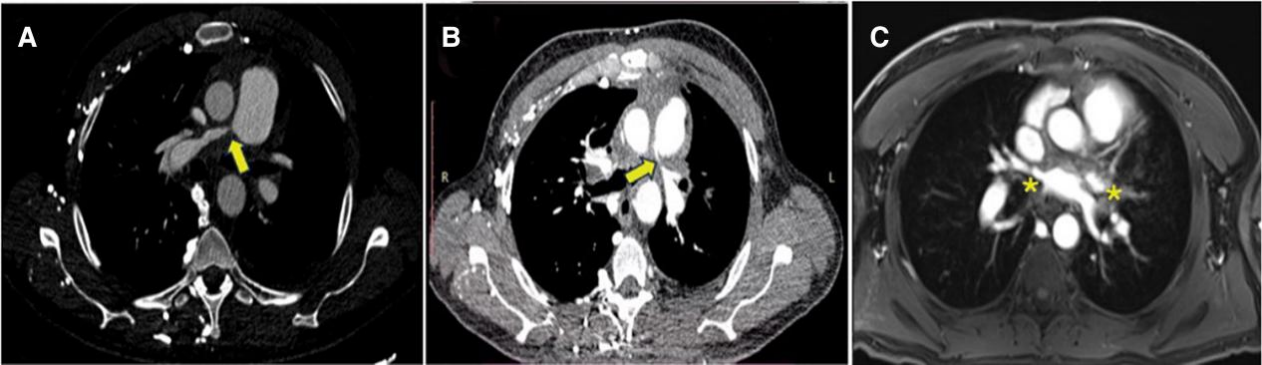
Angiotomography of great vessels and MRI of the thorax. Severe obstruction of the right (*A*) and left (arrows) (*B*) pulmonary arteries is evident. Chest magnetic resonance. (*C*) Scar tissue is observed in the superior mediastinum that involves branches of the right and left pulmonary arteries (asterisks).

**Figure 3:**
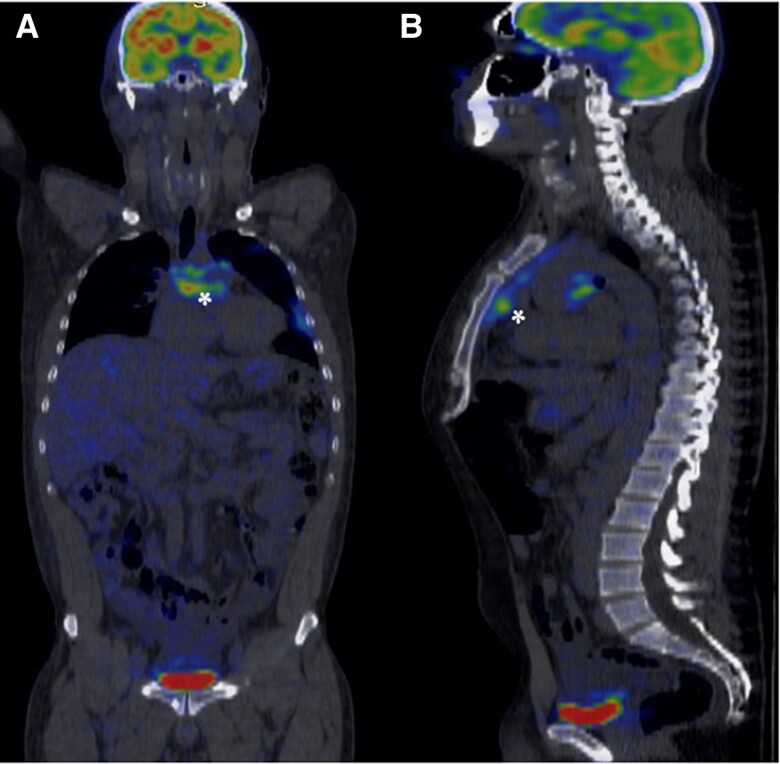
Fluorodeoxyglucose positron emission tomography. Hypermetabolism (asterisks) is observed in irregular lamellar tissue surrounding great vessels (*A*) and the heart (*B*).

During his hospitalization, the ECG showed sinus rhythm, with complete right bundle branch block. Transthoracic echocardiography revealed a dilated right ventricle and atrium, left ventricular ejection fraction (LVEF) of 60%, decreased right ventricular systolic function (TAPSE 15 mm, s′ 8 cm/s) (*Figure 1*), high probability of PH, TRV (tricuspid valve regurgitation) 4 m/s, and PASP (pulmonary artery systolic pressure) 85 mmHg with evidence of supravalvular stenosis (see Supplementary material online, *Figure S2*). There was no evidence of carcinoid valvulopathy, but there was tricuspid annular dilation. Cardiac tomography confirmed extrinsic compression (*Figure 2A* and *B*). MRI (magnetic resonance image) of the thorax showed signs of mild fibrotic changes in the anterior and middle mediastinum with vascular compromise without evidence of a mediastinal mass (*Figure 2C*). Fluorodeoxyglucose positron emission tomography (FDG-PET) demonstrated hypermetabolism in the anterior mediastinum, middle, and pleural level (*Figure 3*)

**Figure 4:**
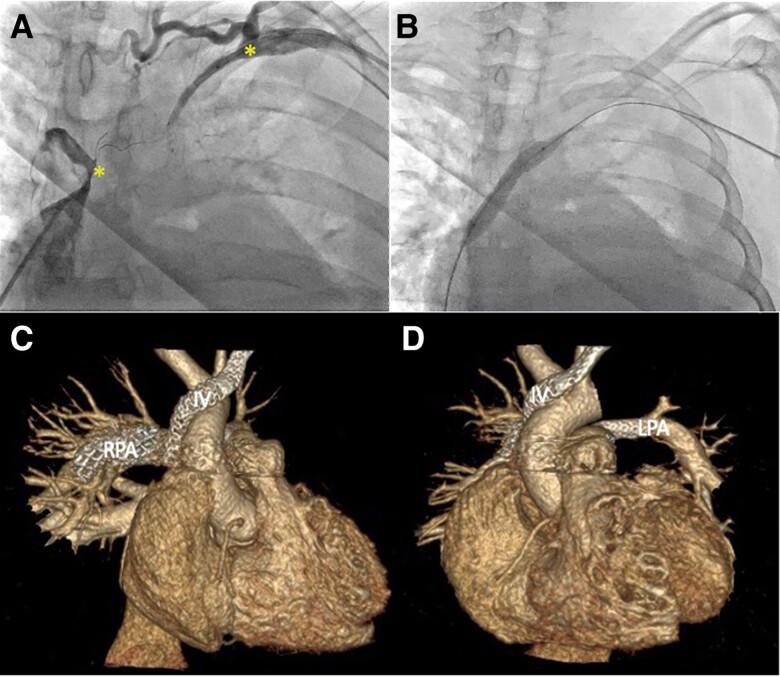
Venous angiography. (*A*) shows total obstruction at the level of the superior vena cava and the innominate vein (asterisks). (*B*) Balloon dilation at the level of the innominate vein. Three-dimensional reconstruction of cardiac computed tomography. (*C*) Image shows stent in the right pulmonary artery (RPA) and innominate vein (IV). (*D*) A stent is evident in the left pulmonary artery (LPA) and IV.

During hospitalization, the patient developed superior vena cava syndrome, prompting a venography that was performed that revealed a total obstruction of the superior vena cava before its junction with the azygos vein and the innominate vein (*Figure 4A*). Right heart catheterization indicated PH with a mean pulmonary arterial pressure of 38 mmHg. The flow in the left innominate vein was restored through percutaneous transluminal balloon dilation (*Figure 4B*). One week later, percutaneous endovascular treatment was performed with stent placement in the left pulmonary artery (01), the right pulmonary artery (03), and the innominate vein (01) (*Figure 4C* and *D*). During the procedure, a pericardial effusion was observed without echocardiographic signs of pericardial tamponade, which was managed conservatively, resulting in improvement over the following 7 days.

Given the suspicion of a possible neoplasia in the area showing metabolic activity on FDG-PET (*Figure 3*), pleural biopsy was performed via thoracoscopy. However, no malignant neoplasia was evident, leading to initial diagnosis of fibrosing mediastinitis of unknown aetiology, after ruling out the primary causes. Medical treatment for PH was considered, focusing on correcting structural abnormalities and using stents to alleviate vascular obstructions. Reduction in PASP was secondary to mechanical relief of vascular compression.

**Figure 5:**
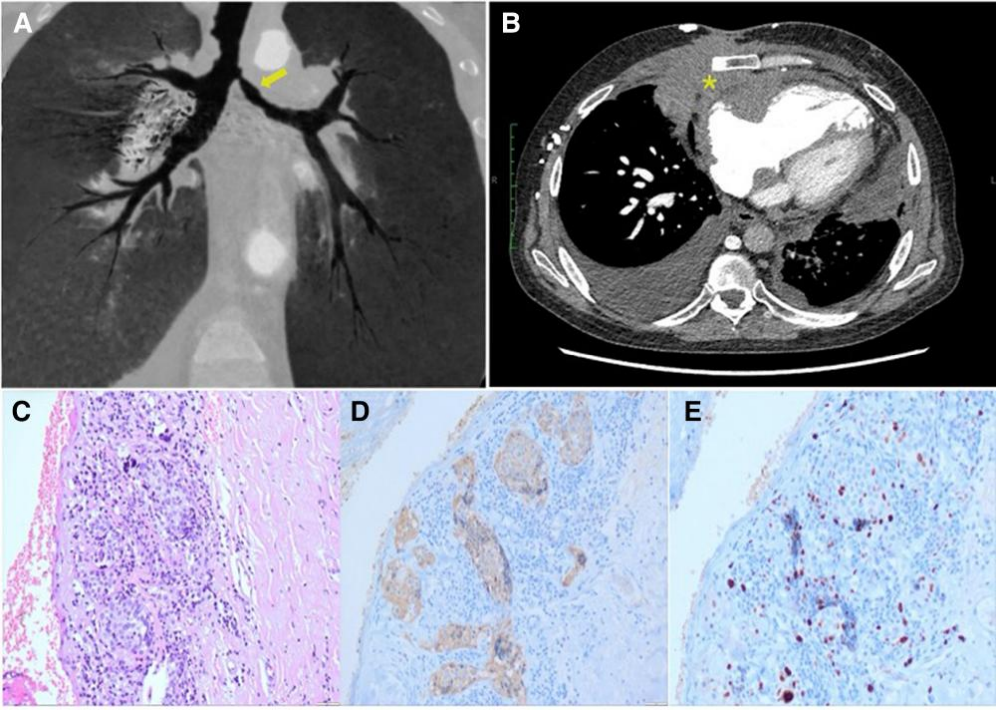
Chest computed tomography. (*A*) A 70% stenosis of the left bronchus lumen is observed (arrow). (*B*) Anterior mediastinal mass with bone and right ventricular involvement (asterisk). Pathological anatomy. (*C*) Haematoxylin eosin stain shows pericardial tissue infiltrated by well-differentiated neuroendocrine tumour nests. (*D*) Immunohistochemistry is positive for synaptophysin. (*E*) Proliferation index with Ki-67 of 20%.

The patient was discharged and followed up as an outpatient with chest computed tomography scan after 18 months, which showed adequate patency of the stents but revealed 70% obstruction of the left bronchus (*Figure 5A*). Additionally, an anterior mediastinal mass was identified (*Figure 5B*), prompting hospitalization for a biopsy (*Figure 5C-5E*), which revealed the following immunohistochemistry results: chromogranin: positive, synaptophysin: positive, pankeratin: positive, CD45: negative, and Ki-67: 20%. This was compatible with atypical thymic carcinoid.

Chemotherapy treatment with agents such as capecitabine, temozolomide, cisplatin, and etoposide was decided due to the diffuse involvement of the mediastinum. The focus was on cytotoxic chemotherapy rather than targeting the serotonin pathway due to the absence of clinical symptoms such as diarrhoea, flushing, abdominal pain, tricuspid valvulopathy, or bronchial obstruction. Tumour resection was not considered due to the extent and invasiveness of the mass, and radiotherapy was not deemed a viable option. No improvement was observed in subsequent FDG-PET scans. Follow-up echocardiographic showed a decrease in LVEF to 40% with persistent right ventricular dysfunction. The patient passed away 48 months after the onset of symptoms of PH, secondary to pulmonary infection at the referring hospital.

## Discussion

Pulmonary hypertension secondary to extrinsic compression of the pulmonary vasculature by a mediastinal tumour is a rare condition that presents a significant diagnostic challenge. Malignant mediastinal tumours can induce PH through various mechanisms, with extrinsic vascular compression being the likely cause in this case.

Neuroendocrine tumours are epithelial neoplasms with predominant neuroendocrine differentiation that can arise in many organs, including the lung, thymus, gastrointestinal tract, and ovary.^9^ Thymic NETs are classified as typical carcinoids, atypical carcinoids (AC), large cell neuroendocrine carcinomas, and small cell carcinomas.^10^ Among these, AC are the most common, typically presenting between the ages of 40 and 50. Patients may remain asymptomatic, but symptoms can occur due to compression or invasion of adjacent intrathoracic structures or the tumour’s ability to produce hormones^11^ Unlike other types of neuroendocrine neoplasms, in patients with thymic carcinoids, a carcinoid syndrome is <1%.^12^ Treatment of thymic NETs involves definitive oncological resection of the primary tumour, which is currently the only option with the potential for a long-term cure. Systemic management for this pathology is not yet well-defined.^6^

This case highlights the importance of considering PH of oncological aetiology as a possible cause of progressive dyspnoea. Furthermore, it highlights the need for a comprehensive evaluation, including imaging and biopsy, to establish an accurate diagnosis and guide early treatment. Despite early detection and intervention, the prognosis of these patients remains poor.

## Lead author biography


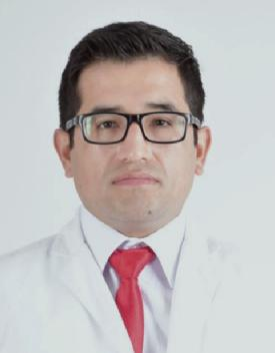


Dr Maicol Cortez is an associate physician in the Cardiology Department at Edgardo Rebagliati Martins National Hospital. His areas of interest include electrophysiology and cardiac imaging.

## Supplementary Material

ytae678_Supplementary_Data

## Data Availability

The data underlying this article are available to use for all readers.

## Supplementary material

**Consent:** The authors confirm that written consent for the submission and publication of this case report, including images and associated text, has been obtained from the patient in accordance with COPE guidance.

**Funding:** There is no funding source for this study.
