# Supplementary material for: When the heart and tumours intertwine: pulmonary hypertension associated with a neuroendocrine tumour—a case report
Source: Eur Heart J Case Rep. 2024 Dec 31;9(1):ytae678. doi: 10.1093/ehjcr/ytae678 (PMC11748130; doi:10.1093/ehjcr/ytae678)
Supplement: ytae678_Supplementary_Data [file ytae678_supplementary_data.docx]

**
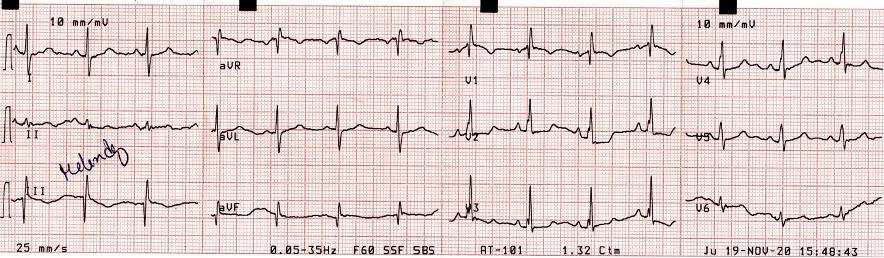
**

Figure S1. Electrocardiogram showed sinus rhythm, borderline right axis deviation (+90°) and S1Q3T3 pattern.

**
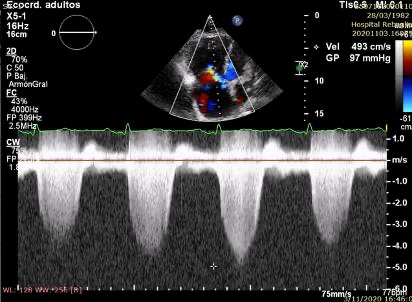
**

Figure S2. Transthoracic echocardiography revealed high probability of PH (TRV 4 m/s) and PSAP 85mmHg with evidence of supravalvular stenosis.
